# Effect of malnutrition on radiographic findings and mycobacterial burden in pulmonary tuberculosis

**DOI:** 10.1371/journal.pone.0214011

**Published:** 2019-03-27

**Authors:** Kacie J. Hoyt, Sonali Sarkar, Laura White, Noyal Mariya Joseph, Padmini Salgame, Subitha Lakshminarayanan, Muthuraj Muthaiah, Saka Vinod Kumar, Jerrold J. Ellner, Gautam Roy, C. Robert Horsburgh, Natasha S. Hochberg

**Affiliations:** 1 Department of Epidemiology, Boston University, Boston, MA, United States of America; 2 Department of Preventive and Social Medicine, JIPMER, Puducherry, India; 3 Department of Biostatistics, Boston University, Boston, MA, United States of America; 4 Department of Microbiology, JIPMER, Puducherry, India; 5 Rutgers—New Jersey Medical School, Newark, NJ, United States of America; 6 Intermediate Reference Laboratory, Government Hospital for Chest Diseases, Puducherry, India; 7 Department of Pulmonary Medicine, JIPMER, Puducherry, India; 8 Department of Medicine, Section of Infectious Diseases, Boston University School of Medicine, Boston, MA, United States of America; Karolinska Institutet, SWEDEN

## Abstract

**Background:**

The relationship between malnutrition and tuberculosis (TB) severity is understudied. We investigated the effect of malnutrition on radiographic findings and mycobacterial burden.

**Methods:**

Subjects included newly diagnosed, smear-positive, culture-confirmed, pulmonary TB cases enrolled in the Regional Prospective Observational Research for TB (RePORT) cohort. Multivariate regression models were used to evaluate the relationship at start of treatment between body mass index (BMI) and chest radiograph (CXR) findings of cavitation and percentage of lung affected and mycobacterial growth indicator tube (MGIT) time to positive (TTP). Severe malnutrition was defined as BMI<16 kg/m^2^, moderate malnutrition as 16–18.4kg/m^2^, and “normal”/overweight as ≥18.5 kg/m^2^.

**Results:**

Of 173 TB cases with chest x-ray data, 131 (76%) were male. The median age was 45 years (range 16–82); 42 (24%) had severe malnutrition and 58 (34%) moderate malnutrition. Median percentage of lung affected was 32% (range 0–95), and 132 (76%) had cavitation. Individuals with severe malnutrition had, on average, 11.1% [95% CI: 4.0–13.3] more lung affected, compared to those with normal BMI, controlling for diabetes and cavitation. In multivariable analyses, cases with severe malnutrition had a 4.6-fold [95% CI, 1.5–14.1] increased odds of cavitation compared to those with normal BMI, controlling for smoking. Median MGIT TTP was 194.5 hours. Neither severe (aRR 0.99; 95% CI, 0.9–1.2) nor moderate (aRR 0.97; 95% CI, 0.8–1.1) malnutrition was associated with MGIT TTP.

**Conclusion:**

We found that malnutrition was associated with increased extent of disease and cavitation on CXR. These findings may reflect the immunomodulatory effect of malnutrition on pulmonary pathology.

## Introduction

The World Health Organization states that India had 2.7 million cases of tuberculosis (TB) in 2017, accounting for 27% of cases globally [1]. Malnutrition is also prevalent in much of India; among adults age 15–49 years, 34% of men and 36% of women have malnutrition defined as body mass index (BMI) <18.5 kg/*m*^2^ [2]. These colliding epidemics are of particular concern because malnutrition is associated with more than an 8-fold increased risk of pulmonary TB (PTB), compared to BMI ≥ 18.5 kg/*m*^2^ [3]. A study among 2 cohorts in Taiwan reported 2-fold increase in TB risk among underweight subjects and 67% and 64% reductions in tuberculosis hazards among obese subjects [4]. Similarly, a meta-analysis of 6 studies found a log-linear dose-response relationship between BMI and TB incidence and a 13.8% decrease in TB incidence per unit increase in BMI [5]. In southern India, 61.5% of TB cases in women and 57.4% in men are attributable to malnutrition [6].

A clear association between malnutrition and TB risk exists, but less is known about the impact of malnutrition on TB severity, and data are conflicting. An Ethiopian study found that among 83 malnourished, HIV- uninfected subjects, BMI < 16 was associated with decreased odds of cavitation controlling for age, gender, and area of residence [aOR 0.65; 95% CI 0.48–0.88], but did not control for mycobacterial burden or duration of illness in this model [7]. By contrast, a Latvian study among 995 adult multi-drug resistant (MDR)-TB patients reported BMI <18.5 was associated with bilateral cavitation, controlling for age [aOR 2.1; 95% CI 1.3–3.5]; however, these findings may not apply to new TB cases [8]. In Malawi, BMI <19 was associated with far advanced lung disease (aOR 8.83; 95% CI 3.64–21.42), but the study did not assess cavitation and did not control for duration of illness which might be associated with weight loss and low BMI [9]. Hence the association between malnutrition and TB radiographic findings have been inconclusive and limited by not controlling for important confounders.

Studies of the effect of malnutrition on mycobacterial burden are similarly limited. Analysis of data from the Latvian national MDR-TB database found that malnourished individuals >18 years of age had 2.7 times the odds of having a 3+ grade culture compared to those that had normal or overweight BMI, but multivariable analyses were not done [8]. Small mid-upper arm circumference (MUAC) was significantly associated with increased AFB grade in Tanzania, controlling for sociodemographic factors, but not comorbidities or duration of illness [10]. To our knowledge, studies have not looked at the impact of malnutrition on mycobacterial burden as measured by mycobacterial growth indicator tube (MGIT).

Understanding how malnutrition affects radiographic findings and mycobacterial burden is important, as these factors affect transmissibility to others and predict treatment outcomes and long-term pulmonary damage [8,11,12]. Further delineation of the role malnutrition plays in TB disease severity may also clarify whether malnutrition affects TB treatment response. The objective of this analysis was to investigate the effect of malnutrition on CXR findings and mycobacterial burden in HIV-uninfected pulmonary TB patients. This study addressed limitations of previous studies by controlling for previously overlooked confounders of the association.

## Materials and methods

### Study population and design

A secondary analysis was conducted using data collected for the Indo-US Regional Prospective Observational Research in TB (RePORT) study. Details of the study design have been previously reported [6,11]. In brief, new smear-positive TB suspects identified by the Revised National TB Control Program (RNCTP), that received <1 week of TB treatment were enrolled in Puducherry and two districts of Tamil Nadu: Villupuram and Cuddalore. Those with known MDR-TB or known contact with an MDR-TB case were excluded and those without growth on culture were retrospectively excluded. Tuberculosis cases were assessed at enrollment with a sputum smear, liquid culture (MGIT), demographic and clinical questionnaire, and anthropometric measurements including BMI. Starting in January 2015, chest radiographs (CXR) were performed. Each radiograph was scored independently by two trained CXR readers using a standardized form. The form first rated the quality of 3 (anterior-post, lateral, and postero-anterior) views as: acceptable; poor, but readable; not acceptable/readable. Abnormal CXRs were evaluated for presence/absence of cavitation and opacity in the upper zone, mid zone, and lower zone independently. Mediastinal adenopathy, pleural effusion, hilar adenopathy, bronchiectasis, and collapsed lung were also indicated as present or absent. Based on these findings, each reader assigned an overall percentage of lung affected. Cavitation was not included in the measure of percentage of lung affected, but considered an independent, dichotomous measure of severity. Findings were discussed until a consensus was reached on the final score. This analysis was restricted to subjects with CXR data enrolled from January 2015 to August 2017; those with HIV infection (n = 6) and age <15 years (n = 3) were excluded.

### Variable definitions

Severe malnutrition was categorized as BMI <16 kg/*m*^2^, malnutrition as BMI 16–18.4, and normal/overweight ≥18.5, as per WHO categorization [13]. Risky alcohol use was determined using Alcohol Use Disorders Identification Test-Consumption (AUDIT-C), a validated score that consists of questions to classify hazardous patterns of alcohol use (≥8 is risky) [14]. Diabetes was defined as random blood sugar >200mg/dL or known diagnosis of diabetes. Maximum symptom duration was the longest period a subject reported any symptom of TB. Years of education was dichotomized into those that completed primary education or less (≤9 years) and those that finished more.

### Statistical analysis and ethical approval

Chi-square and ANOVA tests were conducted to determine associations between variables and BMI. Univariate and multivariable linear, logistic, and negative binomial regression models were used to determine unadjusted and adjusted associations between descriptive variables and percentage of lung affected, cavitation, and MGIT, respectively. AIC was used to select between negative binomial and Poisson regression in the MGIT univariate and multivariate models. Model building was performed by including covariates with p≤ 0.2 from univariate analysis into the model; a backward model-building approach was used (variables resulting in ≥10% change in estimate were retained.) All data analyses were conducted using SAS 9.4 (SAS Institute, Cary, NC). The study was approved by the Institutional Review Boards of Boston University and Rutgers–New Jersey Medical School, and the Jawaharlal Institute of Postgraduate Medical Education and Research (JIPMER) Ethics and Scientific Advisory Committees.

## Results

### Characterization of study population

Analyses were conducted on 173 subjects with CXR data. Subjects in this subset had similar characteristics to those without CXRs (S1 Table). Males accounted for 131 (76%), and the median age was 45 years (range 16–82). Of the 173 subjects, 42 (24%) had severe malnutrition, 58 (34%) had moderate malnutrition, and 73 (42%) were normal/overweight (including 5 (2.9%) who were overweight). Cough for more than 4 weeks was reported by 126 (73%). Risky alcohol use was reported by 81 (47%), and 80 (46%) were current/former smokers. Cavitation was present on 132 (76%) of chest x-rays, and the median percentage of lung affected was 32% (range 0–95%).

### Characteristics of cases with malnutrition

Although age, sex, years of education, cough duration, and overall symptom duration did not differ significantly between subjects with severe malnutrition, moderate malnutrition and normal BMI, those that had severe malnutrition were more likely to smoke (26/42 [62%]) compared to those with moderate malnutrition (26/58 [45%]) or normal/overweight BMI (28/73 [38%]; p = 0.07; Table 1). Similarly, subjects with severe malnutrition were more likely to report risky alcohol use (25/42 [60%]) compared to those with malnutrition and normal BMI (28/58 [48%] and 28/73 [38%] respectively; p = 0.09). Severely malnourished cases were also significantly less likely to have diabetes than malnourished or normal cases (10%, 24%, and 48%, respectively; p<0.0001).

**Table 1:** Characteristics of pulmonary TB cases stratified by body mass index (BMI), n = 173.

	Total	Severe Malnutrition	Malnutrition	Normal/ Overweight	P values for univariate association with BMI
		(n = 42)	(n = 58)	(n = 73)	
**Gender, n (%)**					
Male	131 (75.7)	33 (78.6)	42 (72.4)	56 (76.7)	p = 0.75
Female	42 (24.3)	9 (21.4)	16 (27.6)	17 (23.3)	
**Median age, years (range)**	45(16–82)	41.5 (18–71)	44 (16–81)	46 (21–82)	p = 0.15
**Years of education, n (%)**					
9 years or less	115 (66.5)	28 (66.7)	36 (62.1)	51 (69.9)	p = 0.64
>9 years	58 (33.5)	14 (33.3)	22 (37.9)	22 (30.1)	
**COPD/asthma, n (%)**					
Yes	5 (2.9)	2 (4.8)	2 (3.4)	1 (1.4)	p = 0.55
No	168 (97.1)	40 (95.2)	56 (96.6)	72 (98.6)	
**Diabetes mellitus, n (%)**					
Yes	53 (30.6)	4 (9.5)	14 (24.1)	35 (48.0)	p<0.0001
No	120 (69.4)	38 (90.5)	44 (75.9)	38 (52.1)	
^a^**Risky alcohol use, n (%)**					
Yes	81 (46.8)	25 (59.5)	28 (48.3)	28 (38.4)	p = 0.09
No	92 (53.2)	17 (40.5)	30 (51.7)	45 (61.6)	
**Smoking, n (%)**					
Yes (current)	47 (27.2)	17 (40.5)	17 (29.3)	13 (17.8)	p = 0.07
Yes (former)	33 (19.1)	9 (21.4)	9 (15.5)	15 (20.6)	
No (never)	93 (53.8)	16 (38.1)	32 (55.2)	45 (61.6)	
**Cough duration, n (%)**					
≥4weeks	126 (72.8)	33 (78.6)	36 (62.1)	57 (78.1)	p = 0.08
<4 weeks	47 (27.2)	9 (21.4)	22 (37.9)	16 (21.9)	
**Maximum symptom duration, weeks (range)**	4 (1,24)	4 (2, 24)	4 (1, 12)	4 (1, 24)	p = 0.20
**Median MGIT TTP**^b^**, hours (range)**	194 (39, 1008)	205 (60, 355)	185 (48, 511)	199 (39, 1008)	p = 0.75
**Cavitation, n (%)**					
Yes	132 (76.3)	37 (88.1)	45 (77.6)	50 (68.5)	p = 0.06
No	41 (23.7)	5 (11.9)	13 (22.4)	23 (31.5)	
**Median percent lung affected (range)**	32 (0, 95)	45.5 (5, 93)	30 (8, 95)	24 (0, 89)	p<0.0001

^a^Risky alcohol use based on AUDIT-C score.

^b^MGIT TTP = mycobacterial growth indicator tube time to positive

### Radiographic findings

On univariate analysis, those with severe malnutrition were more likely to have cavitation (OR 3.4; 95% CI 1.2–9.8; Table 2). Current smoking was also associated with cavitation in univariate analysis (OR = 0.5; 95% CI 0.2–1.2). Other factors including diabetes were not associated with cavitation in univariate analysis. In adjusted multivariable analyses, subjects with severe malnutrition were more likely to have cavitation (aOR 4.6; 95% CI 1.5–14.1) than those with normal BMI, controlling for smoking (Table 3). This effect was not seen for those with moderate malnutrition (OR = 1.9; 95% CI 0.8–4.3). In multivariate analysis, current smoking was associated with less cavitation (aOR 0.4; 95% CI 0.2, 0.9).

**Table 2:** Univariate predictors of relative percentage of lung affected, cavitation, and mycobacterial burden, Pondicherry and Tamil Nadu, India (n = 173).

	Univariate OR for cavitation (95%CI)	Univariate relative percentage of lung affected (95%CI)	Univariate RR for MGIT (TTP)^a^ (95%CI)
**BMI**			
Severe malnutrition	3.4 (1.2, 9.8)	16.2 (9.2,23.3)	0.95 (0.8, 1.1)
(BMI < 16kg/m2)	p = 0.06	p<0.0001	p = 0.52
Malnutrition	1.6 (0.7,3.5)	5.2 (-1.2,11.6)	0.94 (0.82,1.1)
(16< = BMI <18.5)	p = 0.72	p = 0.11	p = 0.42
Normal/ Overweight	Ref	Ref	Ref
(BMI ≥18.5)			
**Sex**			
Male	1.4(0.6,3.1)	5.8 (-1.0,12.5)	0.93 (0.8,1.1)
	p = 0.39	p = 0.09	p = 0.33
Female	Ref	Ref	Ref
**Age, years**	1.0 (0.97,1.0)	-0.04 (-0.3,0.2)	0.96 (0.8, 1.2)
	p = 0.41	p = 0.70	p = 0.65
**Years of Education**			
9 years or less	0.9 (0.4, 1.9)	5.5 (-0.6,11.6)	1.1 (0.9, 1.2)
	p = 0.78	p = 0.08	p = 0.19
>9 years	Ref	Ref	Ref
**COPD/Asthma**			
Yes	1.2 (0.13, 11.5)	3.0 (-14.4, 20.5)	0.8 (0.6,1.2)
	p = 0.84	p = 0.73	p = 0.28
No	Ref	Ref	Ref
**Diabetes mellitus**			
Yes	0.7 (0.3, 1.5)	-10.8 (-16.9,-4.7)	1.1 (1.0,1.3)
	p = 0.35	p = 0.0005	p = 0.11
No	Ref	Ref	Ref
**Risky alcohol use**^b^			
Yes	1.0 (0.5, 2.0)	6.8 (1.0, 12.6)	0.9 (0.8, 1.0)
	p = 0.94	p = 0.02	p = 0.23
No	Ref	Ref	Ref
**Smoking**			
Yes (current)	0.5 (0.2, 1.2)	4.2 (-2.6, 11.0)	0.8 (0.7, 0.9)
	p = 0.04	p = 0.22	p = 0.006
Yes (former)	1.5 (0.5, 4.5)	9.0 (1.3, 16.6)	0.98 (0.8,1.2)
	p = 0.16	p = 0.02	p = 0.84
No (never)	Ref	Ref	Ref
**Cough**			
≥4 weeks	1.0 (0.4, 2.2)	1.4 (-5.2, 7.9)	0.95 (0.8,1.1)
	p = 0.96	p = 0.68	p = 0.44
<4 weeks	Ref	Ref	Ref
**Maximum symptom duration, weeks**	1.0 (0.9, 1.1)	0.08 (-0.9, 1.0)	0.99 (0.98,1.0)
	p = 0.99	p = 0.88	p = 0.88
**MGIT TTP, hours**	1.0 (0.9, 1.0)	-0.03 (-0.1, -0.001)	
	p = 0.33	p = 0.04	
**Percent lung affected**	1.06 (1.03,1.08)		0.996(0.99,1.0)
	p<0.0001		p = 0.01
**Cavitation**			
Yes		14.6 (8.1,21.1)	1.1 (0.9,1.3)
		p<0.0001	p = 0.23
No		Ref	Ref

^a^MGIT TTP = mycobacterial growth indicator tube time to positive.

^b^Risky alcohol use based on AUDIT-C score.

**Table 3:** Multivariable models of the effect of body mass index (BMI) on chest x-ray findings of percentage of lung affected (linear regression), cavitation (logistic regression), and mycobacterial burden (negative binomial regression).

	Relative Percentage of Lung Affected		Cavitation		MGIT TTP^a^ (hours)	
	% Change	p-value	OR	p-value	RR	p-value
	(95%CI)		(95% CI)		(95% CI)	
**Malnutrition**						
Severe	11.1	0.002	4.6	0.03	0.99	0.93
	(4.0, 18.3)		(1.5, 14.1)		(0.9, 1.2)	
Moderate	2.4	0.45	1.9	0.75	0.97	0.93
	(-3.8, 8.6)		(0.8, 4.3)		(0.8,1.1)	
Normal/Overweight	Ref		Ref		Ref	
**Diabetes**						
Yes	-7.1	0.02				
	(-13.1,-1.0)					
No	Ref					
**Cavitation**						
Yes	12.2	0.0001				
	(6.0,18.5)					
No	Ref					
**Smoker**						
Current			0.4	0.01	0.8	0.009
			(0.2, 0.9)		(0.7, 1.0)	
Former			0.6	0.12	0.98	0.84
			(0.4, 2.4)		(0.8, 1.2)	
Never			Ref		Ref	

^a^MGIT TTP = mycobacterial growth indicator tube time to positive.

Those with severe malnutrition had on average 16.2% (95% CI 9.2–23.3) more lung affected than those with normal BMI in univariate analysis (Table 2). Male sex (5.8%; 95% CI -1.0–12.5), risky alcohol use (6.8%; 95% CI 1.0–12.6), and former smoking (9%; 95% CI 1.3–16.6) were identified as significant predictors of increased percentage of lung affected and diabetes with less lung affected (-10.8%; 95% CI -16.9–4.7). In multivariable analyses, individuals with severe malnutrition had, on average, 11.1% (95% CI 4.0–8.3) more lung affected, compared to those with normal BMI, controlling for diabetes and cavitation. Moderate malnutrition was not associated with an increase percentage of lung affected (Table 3). In multivariate analyses, diabetes mellitus was associated with decreased percentage of lung affected -7.1 (95% CI -13.1 to -1.0).

### Mycobacterial burden

The median MGIT TTP was 194 hours (range 39–1008); among severely malnourished, malnourished, and normal BMI individuals, the median MGIT TTP was 205, 185, and 199, respectively. Severe and moderate malnutrition were not associated with MGIT TTP in univariate analysis. Current smokers had a decreased risk of a long TTP MGIT (RR 0.8; 95% CI,0.7–0.9, Table 2); hence smokers had a shorter TTP MGIT (greater mycobacterial burden) compared to non-smokers. Percentage of lung affected was not a significant predictor of TTP in a univariate model (RR 1.0; 95% CI 0.99–1.0). No other variables, including cavitation, diabetes, and risky alcohol use were identified as predictors of MGIT. In multivariable negative binomial regression, neither severe (aRR 1.0; 95% CI 0.9–1.2) nor moderate (aRR 1.0; 95% CI 0.8–1.12) malnutrition was associated with MGIT TTP, controlling for smoking status. In multivariate analysis, current smoking was associated with a shorter TTP MGIT (aRR 0.8; 95% CI 0.8, 1.0, Table 3).

## Discussion

This study of new smear-positive, culture-confirmed pulmonary TB cases in southern India evaluated the impact of malnutrition on TB disease severity. Severe malnutrition was significantly associated with a greater percent of lung affected and more cavitation compared to those with normal BMI, controlling for confounders. Neither moderate nor severe malnutrition affected TTP MGIT.

The finding that malnutrition is associated with more extensive radiographic disease likely reflects an immunomodulatory effect of malnutrition. Malnutrition is the leading cause of acquired immunodeficiency and has been labeled nutritionally acquired immune deficiency syndrome [15]. Containment of *Mycobacterium tuberculosis* requires effective innate and adaptive immune responses characterized by a strong T-helper 1 (Th1) response and granuloma formation [16]. In animal models, malnutrition has been linked to reduced lymphocyte counts [17], as well as decreased expression of tumor necrosis factor (TNF), interferon-gamma (IFNγ), and nitric oxide synthase (NOS)-2 which are essential for generation of mycobactericidal nitrogen oxide [18]. Similarly in humans, malnutrition induces T-helper 2 (Th2) and T-regulatory (Treg) cells, skewing away from Th1 [19,20]. It is possible that these combined effects on the immune response alter TB pathogenesis in the setting of malnutrition and lead to more extensive disease and cavitation.

The increased percentage of affected lung that is associated with malnutrition may have implications for chronic sequelae of TB and worsened respiratory health. Studies have shown that more extensive disease on CXR is associated with a decrease in forced expired volume (FEV1) up to 16 years after treatment [21] and that previous TB disease leads to chronic airflow obstruction [22,23]. If the association we found between malnutrition and increased radiographic extent of disease leads to more complications from TB and worse long-term pulmonary health, more attention should be paid to the nutritional status of TB patients. It is possible that nutritional supplementation early in the TB disease course might mitigate these effects; in one small study, macronutrient supplementation was associated with increased bacterial clearance [24]. Such an intervention would need to be weighed against the data from a Cochrane review that found no benefit of macronutrient supplementation for cure or mortality (although the sample size was likely too small to detect an effect) [25].

Our data suggest that diabetes mellitus is associated with decreased percentage of lung affected. Studies have reported atypical radiological findings among diabetics [26,27], whereas others have found no differences between diabetics and non-diabetics [28]. Diabetic subjects with PTB have been reported to have decreased upper lung field and increased lower lung field involvement compared to non-diabetics with PTB. [26]. Given these findings, additional work is needed to define the effects of malnutrition, diabetes and their interaction on TB pathogenesis and CXR manifestations.

The strengths of this study include use of clearly defined data from a prospective cohort enabling adjustment for potential confounders, including tobacco use, among others which have not been controlled for in previous studies. The inclusion of HIV-uninfected, new TB cases allows us to remove the potential impact of HIV and retreatment on chest x-ray findings. The major limitation of this study is that the data are cross-sectional; therefore no inferences about causation can be drawn. It is possible that some of the malnutrition is due to TB itself, however, studies are quite clear that malnutrition is a strong driver of TB risk [5,6,29]. Furthermore by controlling for duration of symptoms, we were able to look more directly at the effect of malnutrition on TB disease manifestations (rather than malnutrition caused by prolonged TB symptoms). Our use of self-reported data (e.g., symptom duration), however, may be affected by participant recall.

Our finding that severe malnutrition is associated with increased cavitation and extent of disease in pulmonary TB underscores the fact that malnutrition needs to be addressed in areas of the world where the conditions are co-prevalent. Future studies are needed to determine if severe malnutrition correlates with worse TB treatment outcomes and if nutritional interventions for malnourished TB patients might improve the radiographic findings. As we move toward the *End TB* goals [30], all potential strategies need to be evaluated and should be targeted according to the needs of the country [31]. Interventions such as nutritional support would be a potential component of this strategy in India and would have major ancillary benefits for health.

## Supporting information

S1 Table Comparison of RePORT cohort with and without CXR results, using chi-square tests of independence and t-tests. ^a^Risky alcohol use based on AUDIT-C score. ^b^MGIT TTP = mycobacterial growth indicator tube time to positive.
(DOCX)

S1 Dataset Dataset used for the current analysis. Sheet one provides the data for the large cohort used for comparison in supplemental table (S1 Table). Sheet two provides the data for the current analysis.
(XLSX)
