# Supplementary material for: Effect of malnutrition on radiographic findings and mycobacterial burden in pulmonary tuberculosis
Source: PLoS One. 2019 Mar 27;14(3):e0214011. doi: 10.1371/journal.pone.0214011 (PMC6436704; doi:10.1371/journal.pone.0214011)
Supplement: S1 Table — aRisky alcohol use based on AUDIT-C score. bMGIT TTP = mycobacterial growth indicator tube time to positive. (DOCX) [file pone.0214011.s001.docx]

**Supplemental Table 1.** Comparison of RePORT cohort with and without CXR results. Groups compared using chi-square tests of independence and t-tests.

|  | **RePORT cohort without CXRs** | **RePORT cohort with CXRs** | **p-value** |
| --- | --- | --- | --- |
|  | **n=603** | **n=173** |  |
| **BMI** |  |  |  |
| Severe malnutrition (BMI < 16kg/m2) | 180 (29.9) | 42 (24.3) | 0.17 |
| Malnutrition | 213 (35.3) | 58 (33.5) |  |
| (16<=BMI <18.5) |  |  |  |
| Normal/ Overweight | 210 (34.8) | 73 (42.2) |  |
| ( BMI ≥18.5) |  |  |  |
| **Gender** |  |  |  |
| Male | 468 (77.6) | 131 (75.7) | 0.60 |
| Female | 135 (22.4) | 42 (24.3) |  |
| **Age, years; median (range)** | 45(15-77) | 45 (16-82) | 0.96 |
| **Years of Education** |  |  |  |
| 9 years or less | 519 (67.1) | 115 (66.5) | 0.39 |
| >9 years | 255 (32.9) | 58 (33.5) |  |
| **COPD/Asthma** |  |  |  |
| Yes | 12 (2.7) | 5 (18.2) | 0.12 |
| No | 591 (97.3) | 22 (81.5) |  |
| **Diabetes mellitus** |  |  |  |
| Yes | 151 (25.3) | 53 (30.6) | 0.16 |
| No | 447 (74.8) | 120 (69.4) |  |
| **Risky alcohol use^a^** |  |  |  |
| Yes | 264 (43.8) | 81 (46.8) | 0.48 |
| No | 339 (56.2) | 92 (53.2) |  |
| **Smoking** |  |  |  |
| Yes (current) | 125 (20.7) | 47 (27.2) | 0.007 |
| Yes (former) | 186 (30.9) | 33 (19.1) |  |
| No (never) | 292 (48.4) | 93 (53.8) |  |
| **Cough** |  |  |  |
| ≥4 weeks | 414 (69.5) | 126 (72.8) | 0.39 |
| <4 weeks | 182 (30.5) | 47 (27.2) |  |
| **Maximum symptom duration, weeks** | 4 [1,36] | 4 [1,24] | 0.05 |
| **MGIT TTP^b^, hours (range)** | 195 [40, 851] | 194 [39, 1008] | 0.67 |

^a^Risky alcohol use based on AUDIT-C score. ^b^MGIT TTP = mycobacterial growth indicator tube time to positive.
